# Novel screening model of obstructive sleep apnea for snorers with suspected NAFLD undergoing liver sonography

**DOI:** 10.1186/s12890-021-01759-1

**Published:** 2021-11-26

**Authors:** Yang-Bor Lu, Yu-Chieh Weng, Yung-Ning Huang, Hsiung-Ying Huang, Pei-Ting Cheng, Hui-Shan Hsieh, Ming-Shao Tsai

**Affiliations:** 1 Department of Digestive Disease, Xiamen Chang Gung Hospital, Xiamen, China; 2 Hepatobiliary and Pancreatic Unit, Xiamen Chang Gung Hospital, Xiamen, China; 3 Department of Pulmonary and Critical Care Medicine, Sleep Center, Xiamen Chang Gung Hospital, Xiamen, China; 4 Formosa Biomedical Technology Corp., Taipei, Taiwan; 5 Department of Otolaryngology–Head and Neck Surgery, Sleep Center, Xiamen Chang Gung Hospital, No. 123 Avenue Xiafei, Haicang District, Xiamen, 361028 Fujian China; 6 Department of Otolaryngology–Head and Neck Surgery, Chiayi Chang Gung Memorial Hospital, No. 6, Sec. W., Jiapu Rd., Puzi City, 613 Chiayi County Taiwan; 7 College of Medicine, Chang Gung University, Taoyuan, Taiwan

**Keywords:** Obstructive sleep apnea, Elastography, Fatty liver, Steatosis, Screen

## Abstract

**Background/aims:**

Given the increased incidence of obstructive sleep apnea (OSA) among patients with nonalcoholic fatty liver disease (NAFLD), noninvasive screening methods are urgently needed to screen for OSA risk in these patients when conducting an office-based assessment of hepatic steatosis. Therefore, we investigated the controlled attenuation parameter (CAP) and hepatic steatosis index (HSI) in patients with and without OSA and developed screening models to detect OSA.

**Methods:**

We retrospectively reviewed the medical records of all adult snorers with suspected NAFLD undergoing liver sonography between June 2017 and June 2020. Records encompassed CAP and HSI data as well as data collected during in-hospital full-night polysomnography. The multivariate logistic regression models were constructed to explore the predictors of OSA risk. Furthermore, model validation was performed based on the medical records corresponding to the July 2020–June 2021 period.

**Results:**

A total of 59 patients were included: 81.4% (48/59) were men, and the mean body mass index (BMI) was 26.4 kg/m^2^. Among the patients, 62.7% (37/59) and 74.6% (44/59) (detected by the HSI and CAP, respectively) had NAFLD, and 78% (46/59) were diagnosed with OSA on the basis of polysomnography. Three screening models based on multivariate analysis were established. The model combining male sex, a BMI of > 24.8, and an HSI of > 38.3 screened for OSA risk the most accurately, with an area under the receiver operating characteristic curve of 0.81 (sensitivity: 78%; specificity: 85%; and positive and negative predictive values: 95% and 52%, respectively) in the modeling cohort. An accuracy of 70.0% was achieved in the validation group.

**Conclusions:**

The combination screening models proposed herein provide a convenient, noninvasive, and rapid screening tool for OSA risk and can be employed while patients receive routine hepatic check-ups. These models can assist physicians in identifying at-risk OSA patients and thus facilitate earlier detection and timely treatment initiation.

**Supplementary Information:**

The online version contains supplementary material available at 10.1186/s12890-021-01759-1.

## Introduction

Obstructive sleep apnea (OSA) is common, with a prevalence of 2%–4% in the general population and 35–45% in those who are obese [1]. In OSA, the upper airway collapses during sleep, which leads to snoring, hypercapnia, and complications related to chronic intermittent hypoxemia (CIH) [2, 3]. OSA is associated with various metabolic dysfunctions, including insulin resistance and visceral obesity [4, 5]. CIH is independently related to dyslipidemia in nonalcoholic fatty liver disease (NAFLD) [6, 7], thus exacerbating NAFLD and advancing liver fibrosis [8].

NAFLD affects one in four people worldwide [9] and comprises a broad spectrum of liver disorders. The obesity epidemic has increased the incidence of NAFLD [10] and the risk of OSA [11]. The prevalence of NAFLD is thus higher in patients with OSA [12]. Accordingly, it is crucial to screen patients with OSA risk for NAFLD to detect the condition at an early stage.

Noninvasive screening tools, including clinical laboratory parameters and transient elastography devices, have been developed to stage liver disease [13, 14]. One study performed transient elastography by using a novel proprietary algorithm called the controlled attenuation parameter (CAP), which was developed to quantify ultrasound attenuation during liver stiffness measurement (LSM) [15]. The CAP provides a numerical value that correlates with the histologic degree of steatosis [16] and has a favorable diagnostic value for chronic liver diseases [17, 18]. The hepatic steatosis index (HSI), calculated on the basis of the serum aspartate aminotransferase (AST)/alanine aminotransferase (ALT) ratio, body mass index (BMI), sex, and presence of diabetes mellitus, has been validated in a large cohort of more than 10,000 patients [19] and adopted as a screening method for NAFLD [20, 21].

Although patients with OSA and metabolic comorbidities may have higher LSM values [22], according to a review of the literature, no study has comprehensively compared the CAP and HSI values between individuals with and without OSA. This study investigated the performance of two NAFLD indices, the CAP and HSI, when applied to patients with and without OSA and developed models for OSA risk screening.

## Materials and methods

### Study design and data source

We retrospectively reviewed medical records from the Department of Digestive Disease at Xiamen Chang Gung Hospital between June 2017 and June 2020. All adult patients (aged ≥ 18 years) with suspected NAFLD who underwent liver sonography and had self-reported snoring with in-hospital full-night polysomnography were identified. To prevent interference in the liver sonography and polysomnography results, we excluded the following individuals: (a) patients who had previously been diagnosed as having OSA for which they had received interventions (surgical or nonsurgical), including continuous positive airway pressure or oral appliance treatment, (b) patients with a history of excessive alcohol consumption (more than 30 g/day for men and more than 20 g/day for women), (c) and patients with a history of viral hepatitis or hepatic cancer [23]. The study protocol was approved by the Institutional Review Board of Xiamen Chang Gung Hospital (approval No. XMCGIRB 2021017), which waived the requirement for informed consent due to the retrospective nature of the study. All procedures were conducted in accordance with the current regulations.

### Anthropometric and laboratory evaluation

Demographic data, such as age, sex, medical history, daily alcohol consumption, and drug usage, were obtained from medical chart review. Patients’ comorbidities, including diabetes mellitus (DM) and hypertension, were also recorded. BMI was calculated as weight in kg/(height in m)^2^, and waist circumference (cm) was measured using a nonelastic measuring tape at the time of overnight polysomnography. The assessment of all laboratory parameters required for HSI calculation, including AST and ALT, was performed on the day of FibroScan or in the preceding week. The HSI was calculated automatically according to the following formula: 8 × (ALT/AST) + BMI (+ 2 if female; + 2 if DM); HSI > 36.0 has been validated as an indication of NAFLD, with a specificity of 92.4% [19].

### Polysomnography

OSA parameters were documented for each patient during in-hospital full-night polysomnography (N7000 Embla, Broomfield, CO, USA). Polysomnography was scored manually by independent investigators according to the standard criteria [24]. The apnea–hypopnea index (AHI) was defined as the sum of all obstructive and mixed apnea (≥ 90% reduction in airflow for a duration of ≥ 2 breaths) plus hypopnea (≥ 50% reduction in airflow, accompanied by ≥ 3% desaturation or electroencephalographic arousal, for a duration of ≥ 2 breaths) divided by the number of hours of total sleep time and the oxygen desaturation index (ODI), which represents the number of events per hour of sleep with ≥ 3% desaturation from baseline [24]. The minimal O_2_ saturation (minimal SpO_2_), mean oxygen saturation (mean SpO_2_), and percentage of total recording time (%TRT) with SpO_2_ < 90% (T90%) were also recorded. OSA was defined as AHI ≥ 5, and the patients with AHI < 5 were included in the non-OSA subgroup.

### CAP

The FibroScan402 equipped with the standard M probe (Echosens, Paris, France) was operated by specially trained nurses according to normal procedures [25] to capture both CAP (dB/m) and LSM (kPa) values simultaneously under fasting conditions for all patients. Examinations with poor reliability, defined as < 10 valid acquisitions or a ratio of the interquartile range (IQR) over the median of 10 measurements (IQR/M) of LSM and CAP > 0.3, were excluded [26]. On the basis of a previous cohort study, a CAP cutoff value of ≥ 238 dm/m was used to determine steatosis grade ≥ S1 (≥ 11% steatosis) [27].

### Validation

Specifically, we extracted medical records from the Department of Digestive Disease corresponding to the July 2020–June 2021 period to perform model validation according to the consistent inclusion and exclusion criteria described previously.

### Statistical analysis

All statistical analyses were completed using SAS version 9.4 (SAS Institute, Cary, North Carolina, USA). Because the preliminary Shapiro–Wilk normality test demonstrated that most variables did not follow a normal distribution, they were presented as the median and IQR and were compared using the Mann–Whitney U test. The chi-square test was used to compare categorical variables between the two groups. Correlations among hepatic indices, BMI, and other OSA severity markers were evaluated using the Spearman correlation. Variables with significant correlations were further subjected to logistic regression analysis. The results of univariate logistic analysis exploring predictors of OSA risk are expressed as odds ratios (ORs) with 95% confidence intervals (CIs). The results of multivariate logistic analysis exploring predictors of OSA risk are expressed as adjusted ORs with 95% CIs. Given that the calculation of HSI takes into account sex, BMI, ALT, AST, and diabetes, sex and BMI may be potential confounders. We further subjected the confounding factors to multivariate logistic regression with the interaction terms HSI × sex and HSI × BMI and found no significant effects. Thus, the confounding effects were not considered in the subsequent combined models. Subsequently, variables with *p* values of < 0.05 in the logistic regression analysis were dichotomized according to the optimal cutoff value by using receiver operating characteristic (ROC) curves [28]. The dichotomized variables were then assessed using multivariate logistic regression to construct combined models. The sensitivity, specificity, positive predictive value (PPV), and negative predictive value (NPV) for each dichotomized value of the predictors and combined models were calculated. The most commonly used index of accuracy was the area under the ROC curve (AUC). The ROC curves were constructed by plotting the sensitivity against (1—specificity) for each combined model. All *p* values were two-sided, and statistical significance was represented by *p* < 0.05.

## Results

### Patients’ characteristics in the modeling group

A total of 94 adult patients with suspected NAFLD and undergoing liver sonography as well as polysomnography were identified. After 35 patients were excluded from the analysis (4 patients had previously been diagnosed as having OSA, 14 patients had a history of excessive alcohol consumption, and 17 patients had a history of viral hepatitis or hepatic cancer), 59 patients were finally enrolled. After the polysomnography reports and medical records of these 59 patients were reviewed, the 46 patients with confirmed OSA (AHI ≥ 5) were designated as the OSA group, and the 13 patients with an AHI of < 5 were designated as the non-OSA group. The patients’ characteristics are presented in Table 1. The OSA group had a male predominance, a significantly higher BMI (*p* < 0.01), and a significantly greater HSI (*p* = 0.03). Moreover, as expected, it had a significantly lower mean SpO_2_, minimal SpO_2_, ODI, and T90(%) (all *p* < 0.01). No significant between-cohort differences in CAP value (*p* = 0.67) or other hepatic indices were observed.

**Table 1:** Baseline clinicodemographic characteristics of the modeling cohort

	All	Non-OSA group	OSA group	*p* value
Patients (n)	59	13	46	
Sex, Males/female	48 (81.4)/11 (18.6)	8 (61.5)/5 (38.5)	40 (87.0)/6 (13.0)	**0.04**
Age (years)	44.3 ± 8.59	42.1 ± 6.78	44.9 ± 9.01	0.30
BMI (kg/m^2^)	26.4 ± 3.0	24.4 ± 3.0	27.0 ± 2.7	** < 0.01**
Waist circumference (cm)	86.3 ± 8.8	82.1 ± 6.6	87.4 ± 9.0	0.05
Diabetes	4 (6.8)	0 (0.0)	4 (8.7)	0.27
Hypertension	14 (23.7)	2 (15.4)	12 (26.1)	0.42
AHI (events/h)	16.7 (5.9–42.2)	1.7 (0.6–3.0)	23.9 (11.3–49.0)	** < 0.01**
Mean SpO_2_ (%)	96.1 (94.0–96.5)	96.5 (96.2–97.0)	95.8 (93.9–96.4)	** < 0.01**
Minimal SpO_2_ (%)	87.0 (80.0–92.0)	93.0 (92.0–94.0)	85.0 (75.0–90.0)	** < 0.01**
ODI (events/h)	12.3 (2.0–29.5)	0.6 (0.0–1.4)	20.1 (8.4–43.3)	** < 0.01**
T90 (%)	0.1 (0.0–1.9)	0.0 (0.0–0.0)	0.3 (0.0–2.9)	** < 0.01**
ALT (IU/L)	32.0 (21.0–44.0)	23.0 (18.0–32.0)	34.0 (22.0–48.0)	0.09
AST (IU/L)	26.0 (20.0–32.0)	24.0 (21.0–27.0)	27.0 (20.0–34.0)	0.17
CAP (dB/m)	269.4 ± 46.4	264.5 ± 47.8	270.8 ± 46.4	0.67
Median	262.0 (236.0–310.0)	243.0 (240.0–304.0)	262.5 (235.0–310.0)	0.71
≥ 238	44 (74.6%)	10 (76.9%)	34 (73.9%)	0.83
HSI	37.4 ± 6.1	34.2 ± 4.5	38.3 ± 6.2	**0.03**
Median	37.5 (33.1–41.0)	33.1 (32.1–37.1)	38.2 (34.3–41.3)	**0.02**
> 36	37 (62.7%)	6 (46.2%)	31 (67.4%)	0.16
LSM (kPa)	5.3 (4.5–6.2)	4.9 (4.1–5.7)	5.6 (4.6–6.7)	0.10

*AHI* apnea–hypopnea index, *ALT* alanine aminotransferase, *AST* aspartate aminotransferase, *BMI* body mass index, *CAP* controlled attenuation parameter, *HSI* hepatic steatosis index, *LSM* liver stiffness measurement, *ODI* oxygen desaturation index, *OSA* obstructive sleep apnea, *SpO*_*2*_ oxygen saturation measured by pulse oximetry, *T90* the percentage of total recording sleep time with oxygen saturation < 90%

Data are summarized as mean ± standard deviation, median (interquartile range), or n (%). Data were compared between non-OSA and OSA groups using the Mann–Whitney U test or chi-square test. Significant *p* values are marked in bold

### Correlations between hepatic indices, BMI, and OSA severity parameters

Significant correlations were observed between the CAP and the following OSA severity indices: AHI (*r* = 0.35, *p* < 0.01), mean SpO_2_ (*r* = −0.35, *p* < 0.01), minimal SpO_2_ (*r* = −0.29, *p* < 0.05), ODI ≥ 3% (ODI3; *r* = 0.33, *p* < 0.01), and T90% (*r* = 0.39, *p* < 0.01; Table 2). The HSI was also significantly associated with AHI (*r* = 0.33, *p* < 0.05) and ODI3 (*r* = 0.26, *p* < 0.05). No other liver parameters had significant correlations with OSA severity indices. BMI was also significantly associated with OSA severity parameters.

**Table 2:** Correlations among hepatic indices, BMI, and OSA severity parameters

	AHI		Mean SpO_2_		Minimal SpO_2_		ODI3		T90%	
	*r*	*P*	*r*	*p*	*r*	*p*	*r*	*p*	*r*	*p*
ALT	0.16	0.24	− 0.03	0.85	− 0.02	0.90	0.02	0.89	0.03	0.84
AST	0.09	0.51	0.05	0.71	0.07	0.60	− 0.04	0.78	− 0.07	0.57
BMI	0.45	** < 0.01**	− 0.34	** < 0.01**	− 0.29	** < 0.05**	0.40	** < 0.01**	0.27	** < 0.05**
CAP	0.35	** < 0.01**	− 0.35	** < 0.01**	− 0.29	** < 0.05**	0.33	** < 0.01**	0.39	** < 0.01**
HSI	0.33	** < 0.05**	− 0.22	0.10	− 0.19	0.15	0.26	** < 0.05**	0.18	0.18

The correlations among the hepatic indices, BMI, and other OSA severity parameters were evaluated using the Spearman correlation. Significant *p* values are marked in bold

*AHI* apnea–hypopnea index, *ALT* alanine aminotransferase, *AST* aspartate aminotransferase, *BMI* body mass index, *CAP* controlled attenuation parameter, *HSI* hepatic steatosis index, *ODI* oxygen desaturation index, *OSA* obstructive sleep apnea, *SpO*_*2*_ oxygen saturation measured by pulse oximetry; T90%, the percentage of total recording time with oxygen saturation < 90%

### Predictors and screening models for OSA risk

From the screening results, we identified predictors of OSA risk (Table 3). Univariate logistic regression revealed that male sex (OR 4.17, 95% CI 1.02–17.05), BMI > 24.8 (OR 1.42, 95% CI 1.09–1.86), and HSI > 38.3 (OR 1.17, 95% CI 1.02–1.36) are significant factors in screening for OSA risk, whereas multivariate logistic regression analysis revealed that male sex (adjusted OR 22.92, 95% CI 1.76–298.92), BMI > 24.8 (adjusted OR 1.83, 95% CI 1.07–3.11), and CAP > 243.5 (adjusted OR 0.96, 95% CI 0.93–0.99) are independent significant predictors of OSA risk. Regarding sex and BMI, the factors with potential confounding effects on HSI, no significant confounding effect was noted in the multivariate logistic regression (*p* = 0.77 and 0.11, respectively). In the subsequent ROC curve analysis, male sex, BMI, the CAP, and the HSI demonstrated fair to acceptable diagnostic performance in screening for OSA risk, with the AUC ranging from 0.54 to 0.75 (Table 4). By using multivariate logistic regression analysis, we constructed the following screening models for OSA risk.

**Table 3:** NAFLD-related parameters derived from the OSA risk screening

	Univariate		Multivariate	
	OR (95% CI)	*p* value	Adjusted OR (95% CI)	*p* value
Male	4.17 (1.02–17.05)	**0.04**	22.92 (1.76–298.92)	**0.02**
Age > 47.5	1.04 (0.96–1.35)	0.29	1.16 (1.00–1.35)	0.05
BMI > 24.8	1.42 (1.09–1.86)	**0.01**	1.83 (1.07–3.11)	**0.03**
CAP > 243.5	1.00 (0.99–1.02)	0.66	0.96 (0.93–0.99)	**0.02**
HSI > 38.3	1.17 (1.02–1.36)	**0.03**	1.33 (0.98–1.80)	0.07

Significant *p* values are marked in bold

*BMI* body mass index, *CAP* controlled attenuation parameter, *CI* confidence interval, *HSI* hepatic steatosis index, *OR* odds ratio

**Table 4:** Screening models for OSA risk

Predictors	Logistic regression		Receiver operating characteristic curve					
	OR (95% CI)	*p* value	Cutoff value	sensitivity	specificity	PPV	NPV	AUC
Original models								
Male	4.2 (1.02–17.05)	**0.047**	Male	87%	39%	83%	46%	0.63
BMI	8.1 (2.06–31.88)	** < 0.01**	> 24.8	78%	69%	90%	47%	0.75
CAP	2.4 (0.69–8.44)	0.17	> 243.5	67%	54%	84%	32%	0.54
HSI	10.1 (1.21–84.03)	**0.03**	> 38.3	46%	92%	96%	32%	0.72
Combined models	Adjusted OR (95% CI)	*p* value	Predictors					
Model A			≥ 3	65%	85%	94%	41%	0.75
Male	2.5 (0.5–12.4)	0.28						
BMI > 24.8	5.3 (1.1–25.6)	**0.04**						
CAP > 243.5	0.6 (0.1–3.0)	0.54						
HSI > 38.3	6.4 (0.7–61.2)	0.11						
Model B			≥ 2	80%	62%	88%	47%	0.71
Male	2.4 (0.5–11.7)	0.28						
BMI > 24.8	7.0 (1.5–33.6)	**0.02**						
CAP > 243.5	0.9 (0.2–4.2)	0.87						
Model C			≥ 2	78%	85%	95%	52%	0.81
Male	2.3 (0.5–11.1)	0.30						
BMI > 24.8	4.5 (1.0–19.4)	**0.045**						
HSI > 38.3	5.6 (0.6–51.9)	0.13						

Significant *p* values are marked in bold

*AUC* area under the curve, *BMI* body mass index, *CAP* controlled attenuation parameter, *CI* confidence interval, *HSI* hepatic steatosis index, *NPV* negative predictive value, *OR* odds ratio, *PPV* positive predictive value

**Fig. 1:**
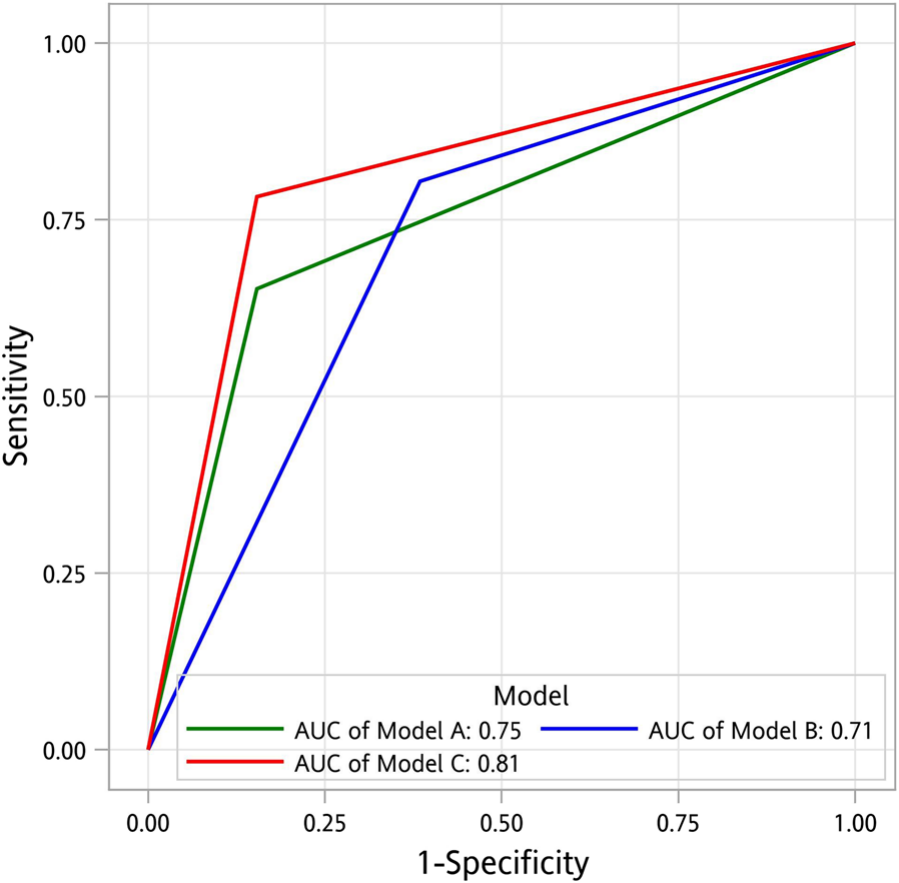
Receiver operating characteristic (ROC) curves of the combined models in the modeling cohort. *AUC* area under the ROC curve

Combined model A included three or more of the following predictors of OSA risk: male sex, BMI > 24.8, CAP > 243.5, and HSI > 38.3; it achieved an AUC of 0.75 (Fig. 1), with a sensitivity of 65%, a specificity of 85%, a PPV of 94%, and NPV of 41%.

Combined model B included two or more of the following predictors of OSA risk: male sex, BMI > 24.8, and CAP > 243.5; it achieved an AUC of 0.71, with a sensitivity of 80%, a specificity of 62%, a PPV of 88%, and NPV of 47%.

Combined model C included two or more of the following predictors of OSA risk: male sex, BMI > 24.8, and HSI > 38.3; it achieved an AUC of 0.81, with a sensitivity of 78%, a specificity of 85%, a PPV of 95%, and NPV of 52%.

### Validation of the screening model for OSA risk

Given the availability of in-hospital polysomnography studies has decreased amid the ongoing COVID-19 pandemic [29] and given some individuals at risk for OSA may not be identified, the sample of 29 patients, which was compared with our modeling cohort. The characteristics of the 29 patients are presented in the Additional file 1: Table S1 and compared with those of the modeling cohort (Additional file 1: Table S2). The screening accuracy of combined models A, B, and C in the validation group was 65.6%, 75.9%, and 70.0%, respectively.

## Discussion

Studies have reported the mutually reinforcing pathogenic effects in NAFLD and OSA [30–32]. Our findings revealed that hepatic steatosis parameters are significantly related to OSA severity and hypoxic burdens, including AHI and ODI3. These findings accord with a previous report that identified a significant correlation between the CAP and the respiratory event index [33]. CIH is a causal factor of NAFLD progression, independent of obesity and metabolic syndrome [34], possibly owing to oxidative stress and mitochondrial dysfunction [35]. However, the roles of various transcription mechanisms of CIH-induced hepatic steatosis remain unclear [33]. In the present study, AHI and ODI were both significant factors related to the CAP and HSI, whereas T90% was not significantly related to the HSI. This finding implies that ODI, reflecting oxygen desaturation frequency, may be more specific for OSA-related CIH than T90%, reflecting hypoxemia severity [22, 36].

Our study also identified the population at high risk for the co-occurrence of NAFLD and OSA. Among patients with NAFLD, 64%–87% have OSA [12, 37–39]; in our study, the prevalence of liver steatosis in the OSA cohort was 73.9% (34/46), a finding that warrants the development of an effective, reproducible, and noninvasive method of screening for OSA risk in this patient population. In the present study, the HSI and CAP both exhibited a high PPV for OSA (96% and 84%, respectively). Furthermore, HSI > 38.3 was more effective in screening for OSA risk than was CAP > 243.5 (AUC: 0.72 and 0.54, respectively), whereas the CAP demonstrated higher sensitivity (67% vs. 46%). CAP ≥ 238 dm/m was reported to indicate hepatic steatosis (> 10% fatty deposit) [27]; the cutoff value of the CAP to screen for OSA risk in our study was somewhat higher (> 243.5 dm/m), implying that OSA worsens hepatic steatosis in individuals with concomitant OSA and NAFLD. HSI > 36.0 was demonstrated to have a 92.4% specificity for detecting NAFLD [19]; in our study, HSI > 38.3 had a 92% specificity for detecting OSA, and the AUC of screening for NAFLD in OSA was similar to that in a previous study [40], but that study reported a lower cutoff value of 35 [40]. This discrepancy might be due to differences in the severity of hepatic steatosis in each cohort. The increased HSI value may reflect the degree of hepatic steatosis, similar to liver echogenicity on ultrasound [19, 41]. Moreover, the higher cutoff value of the HSI in our study might indicate the likelihood of severer steatosis when OSA and fatty liver coexist. Compared with the CAP or HSI alone, the accuracy of screening for OSA risk was higher in the combined models.

The CAP exhibited a lower AUC for screening OSA risk than the HSI not only in the original models (0.54 and 0.72, respectively) but also in the combined models (0.71 and 0.81, respectively), thereby implying that the HSI may be a more accurate tool for screening for OSA risk in individuals with concomitant OSA and NAFLD. The HSI formula uses liver function parameters and demographic characteristics, and thus, it reflects systemic status rather local hepatic elastography, as the CAP does; this approach suggests that OSA is not merely a local event but has further systemic effects [42]. The risk of developing OSA was determined to increase with age and BMI [43] and significant correlations among OSA severity parameters and BMI were also observed in the present study. The fact that BMI was considered in the HSI calculation may explain why the HSI was more accurate in predicting OSA risk in the screening than was the CAP. Although these two hepatic indices differ in screening performance, they are easier to access, provide quantitative results compared with semiquantitative ultrasonography [40], and demonstrate improved accuracy in combination models. The accuracy in the validation group may be affected by the sample size being only half that of the modeling cohort. Nevertheless, we contend that our findings can serve as a reference for clinicians to screen OSA risk in patients with NAFLD more efficiently. The office-based screening models presented in this study may help identify the subpopulation that is more likely to develop OSA before time-consuming examinations such as polysomnography are conducted, thus lowering clinical costs and workloads, especially in places with limited resources. Our simplified screening models use noninvasive and rapid tests that can easily screen patients for OSA risk while surveying hepatic steatosis; thus, they enable the prioritization of at-risk individuals for further assessment and early management. Furthermore, effective treatment of OSA, such as continuous positive airway pressure, can prevent hypoxic events related to upper airway collapse [44], which may stabilize or retard NAFLD progression [38, 45] and hence prevent more hazardous comorbidities.

Our study has some limitations. First, selection bias is possible because we included patients who initially presented to the outpatient clinic in the Department of Digestive Disease for routine check-ups with self-reported snoring. Moreover, we included patients who agreed to undergo in-hospital full-night polysomnography when their physician asked whether they snored. Given that the study cohort did not include patients with a history of hepatic cancer or viral hepatitis and that the combined models were based on NAFLD-related parameters, our models are applicable to individuals with NAFLD. The findings are expected to enhance awareness of sleep apnea among physicians other than sleep specialists, such as hepatologists, who are likely to encounter patients at risk for OSA in daily clinical practice. This simple model can be readily applied in office-based to prioritize early intervention for at-risk patients. Second, liver biopsy data were lacking in this study, the reliability of the CAP varied depending on steatosis grade [46], and the HSI was based on ultrasonography only [19]. Third, our sample size was small; nevertheless, the predictors with AUCs exceeding 0.7 (BMI and HSI) reached a power of ≥ 70%. Furthermore, combined model C reached a power of ≥ 99% and had an acceptable accuracy in the validation cohort. For combined model B, however, theoretically, at least 100 patients must be included to reach a power of ≥ 90%. Further prospective research should employ a larger sample size to optimize the screening models for OSA.

## Conclusions

Significant correlations were observed between the CAP, the HSI, and OSA severity. The HSI seems to be a more accurate indicator than the CAP for OSA risk screening. The models for OSA risk presented in this study may serve as screening tools for individuals receiving routine transabdominal elastography or fatty liver examination. The combined models are applicable to the development of precision medicine protocols for patients with NAFLD and OSA. Furthermore, because of the convenience, noninvasiveness, and rapidity of these screening models, patients suspected of having NAFLD can be screened for OSA risk, thereby facilitating early intervention and management.

## Supplementary Information

**Additional file 1. Table S1**: Baseline clinicodemographic characteristics of the validation cohort. **Table S2**: Baseline clinicodemographic characteristics of validation cohort and modeling cohort.

## Data Availability

All data supporting the finding of this study are available within the manuscript. The datasets generated and/or analyzed during the current study are available from the corresponding author upon reasonable written request.

## Abbreviations

AHI: Apnea–hypopnea index
ALT: Alanine aminotransferase
AST: Aspartate aminotransferase
AUC: Area under the curve
BMI: Body mass index
CAP: Controlled attenuation parameter
CI: Confidence interval
HSI: Hepatic steatosis index
LSM: Liver stiffness measurement
NAFLD: Nonalcoholic fatty liver disease
NPV: Negative predictive value
ODI: Oxygen desaturation index
OR: Odds ratio
OSA: Obstructive sleep apnea
PPV: Positive predictive value
SpO_2_: Oxygen saturation measured by pulse oximetry
T90%: The percentage of total recording sleep time with oxygen saturation < 90%
