# Supplementary material for: Novel screening model of obstructive sleep apnea for snorers with suspected NAFLD undergoing liver sonography
Source: BMC Pulm Med. 2021 Nov 26;21:387. doi: 10.1186/s12890-021-01759-1 (PMC8824741; doi:10.1186/s12890-021-01759-1)
Supplement: Supplementary file 1 — Additional file 1. Table S1: Baseline clinicodemographic characteristics of the validation cohort. Table S2: Baseline clinicodemographic characteristics of validation cohort and modeling cohort. [file 12890_2021_1759_MOESM1_ESM.docx]

**Supplementary table 1.** Baseline clinicodemographic characteristics of the validation cohort.

|  | **All** | **Non-OSA group** | **OSA group** | ***p* value** |
| --- | --- | --- | --- | --- |
| Patients (n) | 29 | 5 | 24 |  |
| Sex, Males/Female | 25 (86.2)/4 (13.8) | 4 (80.0)/1 (20.0) | 21 (87.5)/3 (12.5) | 0.66 |
| Age (years) | 40.4 ± 11.26 | 37.2 ± 5.89 | 41.0 ± 12.07 | 0.50 |
| BMI (kg/m^2^) | 28.3 ± 4.3 | 27.2 ± 1.4 | 28.5 ± 4.7 | 0.73 |
| AHI (events/h) | 22.4 (7.2-58.8) | 2.0 (2.0-3.7) | 31.5 (12.9-63.1) | **<0.01** |
| Mean SpO_2_ (%) | 95.6 (93.8-96.7) | 96.8 (95.4-97.0) | 95.3 (92.9-96.5) | 0.12 |
| Minimal SpO_2_ (%) | 84.0 (79.0-88.0) | 93.0 (90.0-93.0) | 82.0 (76.5-86.0) | **<0.01** |
| ODI (events/h) | 19.6 (6.8-55.8) | 1.5 (0.4-3.0) | 20.8 (9.5-56.9) | **<0.01** |
| ALT (IU/L) | 35.0 (25.0-59.0) | 26.5 (24.0-29.0) | 36.0 (31.0-59.0) | 0.20 |
| AST (IU/L) | 26.5 (21.8-35.0) | 23.0 (20.6-23.0) | 28.0 (23.6-36.0) | 0.19 |
| CAP (dB/m) | 294.4 ± 50.9 | 288.2 ± 32.3 | 295.7 ± 54.4 | 0.51 |
| HSI | 41.7 ± 10.3 | 38.9 ± 2.3 | 42.4 ± 11.4 | 0.80 |

Footnote: a. Data are summarized as mean ± standard deviation, median (interquartile range), or n (%). b. Data were compared between non-OSA and OSA groups using the Mann–Whitney U test or chi-square test. Significant *p* values are marked in bold.

Abbreviations: AHI, apnea–hypopnea index; ALT, alanine aminotransferase; AST, aspartate aminotransferase; BMI, body mass index; CAP, controlled attenuation parameter; HSI, hepatic steatosis index; ODI, oxygen desaturation index; OSA, obstructive sleep apnea; SpO2, oxygen saturation measured by pulse oximetry.

**Supplementary table 2**. Baseline clinicodemographic characteristics of validation cohort and modeling cohort.

|  | **Modeling cohort** | **Validation cohort** | ***p* value** |
| --- | --- | --- | --- |
| Patients (n) | 59 | 29 |  |
| Sex, Males/Female | 48 (81.4)/11 (18.6) | 25 (86.2)/4 (13.8) | 0.57 |
| Age (years) | 44.3 ± 8.59 | 40.4 ± 11.26 | 0.07 |
| BMI (kg/m^2^) | 26.4 ± 3.0 | 28.3 ± 4.3 | 0.11 |
| AHI (events/h) | 16.7 (5.9-42.2) | 22.4 (7.2-58.8) | 0.28 |
| Mean SpO_2_ (%) | 96.1 (94.0-96.5) | 96.5 (93.8-96.7) | 0.42 |
| Minimal SpO_2_ (%) | 87.0 (80.0-92.0) | 84.0 (79.0-88.0) | 0.12 |
| ODI (events/h) | 12.3 (2.0-29.5) | 19.6 (6.8-55.8) | 0.16 |
| ALT (IU/L) | 32.0 (21.0-44.0) | 35.0 (25.0-59.0) | 0.21 |
| AST (IU/L) | 26.0 (20.0-32.0) | 26.5 (21.8-35.0) | 0.66 |
| CAP (dB/m) | 269.4 ± 46.4 | 294.4 ± 50.9 | **0.01** |
| HSI | 37.4 ± 6.1 | 41.7 ± 10.3 | 0.07 |

Footnote: a. Data are summarized as mean ± standard deviation, median (interquartile range), or n (%). b. Data were compared between modeling and validation groups using the Mann–Whitney U test or chi-square test. Significant *p* values are marked in bold.

Abbreviations: AHI, apnea–hypopnea index; ALT, alanine aminotransferase; AST, aspartate aminotransferase; BMI, body mass index; CAP, controlled attenuation parameter; HSI, hepatic steatosis index; ODI, oxygen desaturation index; SpO2, oxygen saturation measured by pulse oximetry.
